# Langerhans Cell Histiocytosis of the Gastrointestinal Tract – A Rare Entity

**DOI:** 10.7759/cureus.2227

**Published:** 2018-02-26

**Authors:** Jasmine Bhinder, Amit Mori, Leon Kurtz, Madhavi Reddy

**Affiliations:** 1 GI, The Brooklyn Hospital Center, Affiliate of the Mount Sinai Hospital. 121 Dekalb Avenue, Brooklyn, Ny 11201; 2 GI, The Brooklyn Hospital Center, Affiliate of the Mount Sinai Hospital. 121, Dekalb Avenue, Brooklyn, Ny 11201; 3 Division of Gastroenterology and Hepatology, Academic Affiliate of the Icahn School of Medicine, Clinical Affiliate of the Mount Sinai Hospital

**Keywords:** histiocytosis, langerhans cell, nodular, gastrointestinal

## Abstract

Langerhans cell histiocytosis (LCH) is an idiopathic and rare disease that ranges in clinical severity based on location and organ involvement. LCH most commonly affects the skin and bones. The involvement of the gastrointestinal tract (GI) in adults is exceedingly rare and only 10 cases have been reported in the literature. We present the case of a 60-year-old male who was referred for a routine screening colonoscopy. Numerous 3-5 mm nodular lesions were present throughout the colon. A histopathological examination revealed diffuse aggregates of histiocytes within the lamina propria of the mucosa and immunohistochemical staining further confirmed the presence of Langerhans cells with a positive CD1-a stain. Although extremely rare, LCH involving the GI tract should be considered as a differential diagnosis when polyps or nodular lesions are witnessed on screening colonoscopies. In addition, the lesions must be biopsied to confirm the diagnosis of LCH and additional follow-up is essential to rule out systemic disease.

## Introduction

Langerhans cell histiocytosis (LCH) is a rare disease of unknown etiology that is characterized by the proliferation of bone-marrow-derived Langerhans cells [1]. The incidence of LCH is most prevalent in childhood and the clinical presentation is highly variable depending on the sites and extent of organ involvement. The multisystem disease most commonly occurs in children, while a more indolent form of the disease localized to a single organ is seen in adults [2]. The most common areas affected include the bone and skin. Other areas involved are the lymph nodes, liver, spleen, lungs, and central nervous system. Involvement of the gastrointestinal tract (GI) is, however, extremely rare. In adults, the prevalence of LCH reported in the GI tract is limited to 10 cases [3].

## Case presentation

We present a case of a 60-year-old male, with a past medical history of chronic obstructive pulmonary disease (COPD), who was referred for a routine screening colonoscopy. He denied any GI complaints prior to the colonoscopy, including abdominal pain, change in bowel habits, blood in stool, or nausea and vomiting. A review of systems was significant for arthralgias present in the knees and elbows and the physical exam was positive for cervical lymphadenopathy. Basic labs, including complete blood count and basic metabolic panel, were within normal limits.

**Figure 1:**
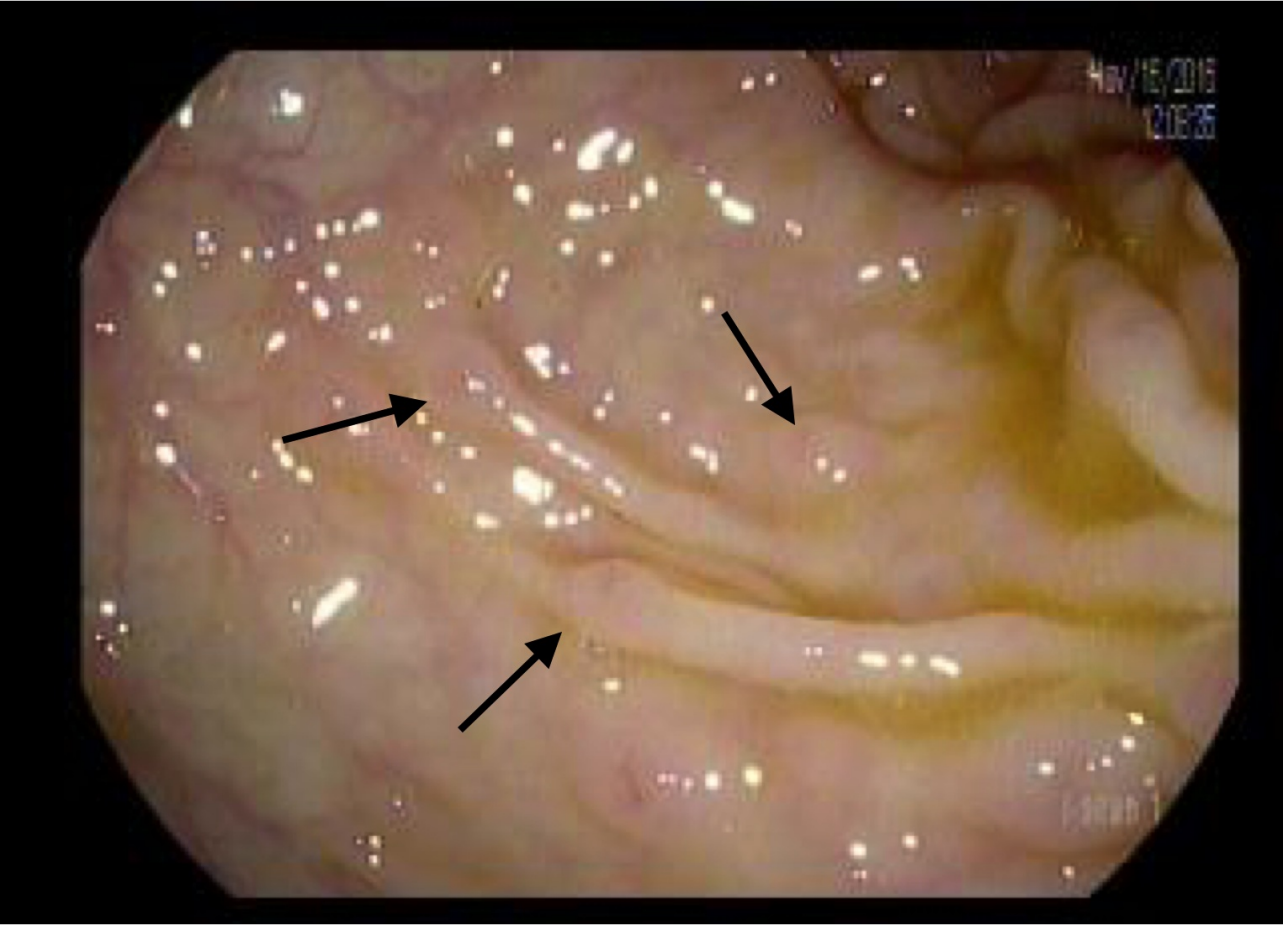
Nodular lesions within the colon

**Figure 2:**
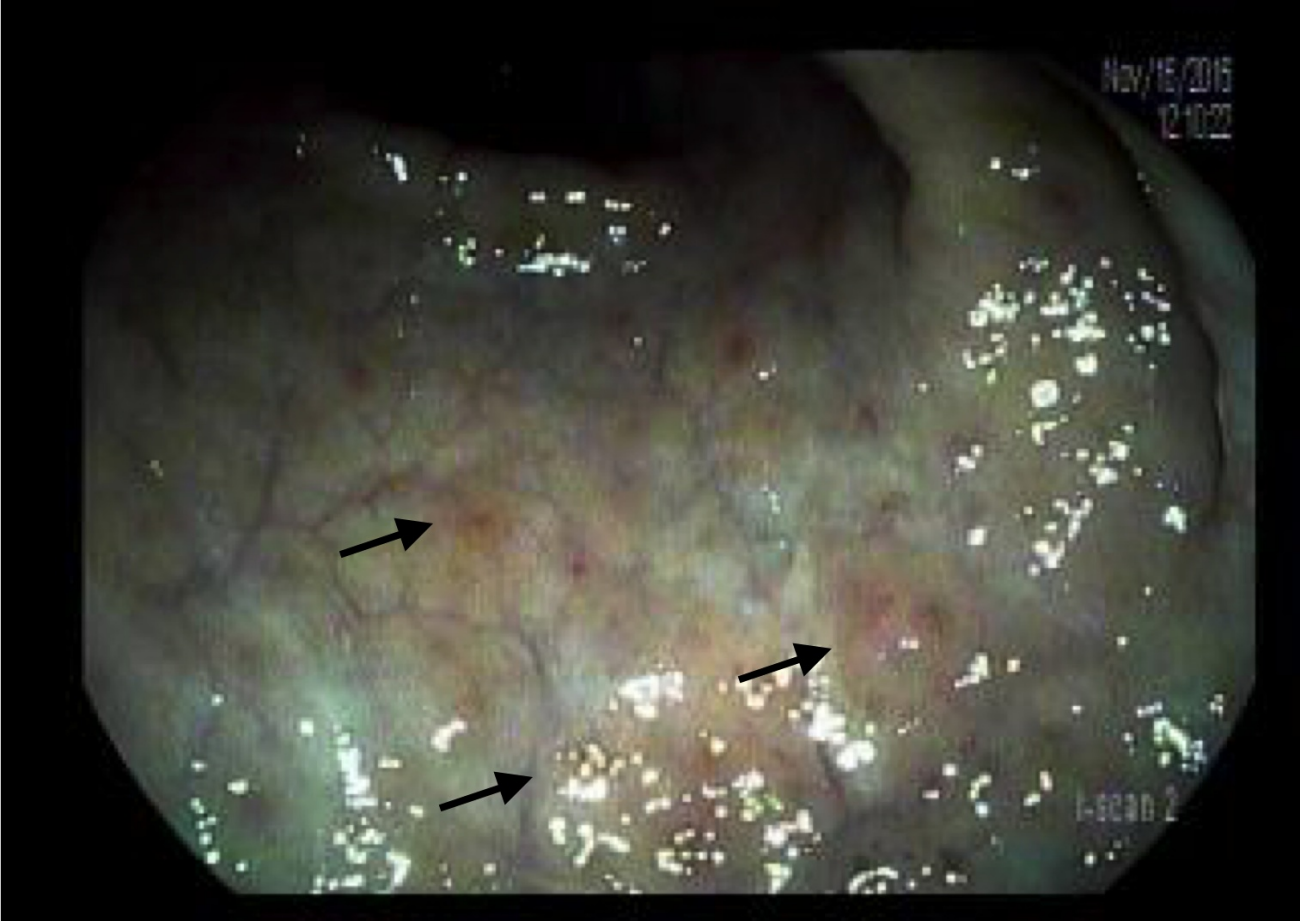
Narrow band imaging (NBI) of nodular lesions

**Figure 3:**
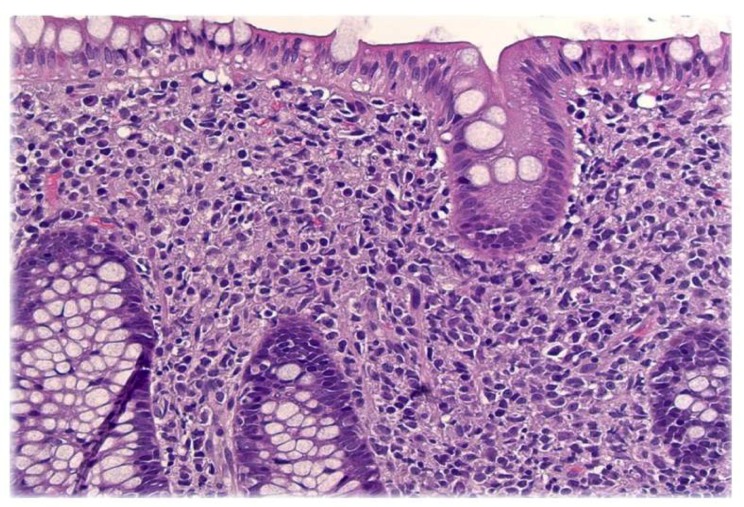
Diffuse aggregates of histiocytes in lamina propria

**Figure 4:**
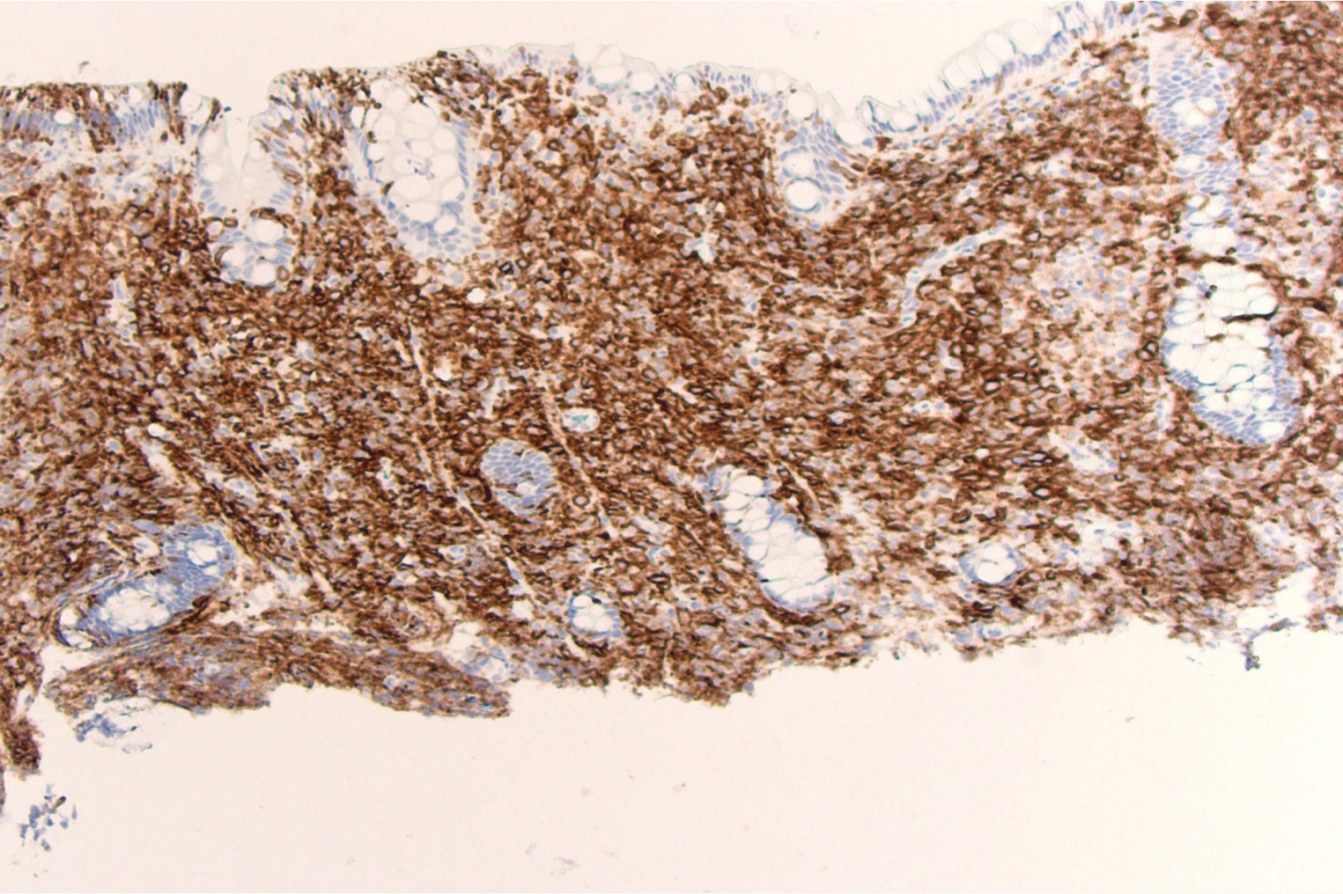
CD-1a immunostaining

Colonoscopy revealed innumerable, 3-5 mm nodular lesions throughout the colon that were more prominent in the transverse and descending colon (Figures 1-2). The nodules were biopsied and a histopathological examination revealed diffuse aggregates of histiocytes within the lamina propria of the mucosa (Figure 3). Immunohistochemical studies further revealed positive staining for CD-1a, confirming the presence of Langerhans cells (Figure 4). An excisional cervical lymph node biopsy was also performed, which revealed T-cell lymphoma. The patient, unfortunately, expired due to complications of the lymphoma a few months later.

## Discussion

Langerhans cells are specialized antigen-presenting cells located predominantly in the skin and mucosa. Their primary function is to engulf and present microbial antigens to T-cells. This generates an active immune response against the microbial invader to fight off infection [1].

Langerhans cell histiocytosis (LCH) is a rare disease characterized by the abnormal proliferation of Langerhans cells that infiltrate multiple organ systems, leading to significant destruction and impairment. The name of this disease is based on the resemblance to Langerhans cells; however, LCH is derived from progenitor cells in the bone marrow and not Langerhans cells of the skin [1]. 

Langerhans cells are also a subtype of histiocyte, which, in general, refers to large white blood cells (WBCs) present in tissues. The term ‘histiocyte’ also includes macrophages and dendritic cells [1.] The World Health Organization of classification of hematopoietic and lymphoid tumors classified disorders of histiocytes into three specific categories, including dendritic cell disorders, macrophage-related disorders, and malignant histiocytic disorders [1]. LCH falls under the first category of these diseases.

LCH has been diagnosed among all age groups; however, it is most commonly seen in children aged 1-3. The incidence is three to five cases per million children, whereas in adults, it is one to two cases per million [4-5]. When comparing pediatric and adult LCH involving the GI tract, the presentation and severity of disease are greatly distinct. LCH in the pediatric population classically presents with symptoms including diarrhea or bloody stools, failure to thrive, abdominal pain, and vomiting [3]. GI involvement occurs most commonly in the context of the multisystem disease, with only a few cases of isolated GI disease being reported. However, in adults, LCH is typically reported as an isolated finding within the GI tract. It characteristically presents as an incidental polyp found on colonoscopy in asymptomatic individuals or patients being evaluated for another reason [3]. In a case series, Singhi et al. described 10 cases of adult LCH involving the GI tract. They reported that five out of 10 adults were asymptomatic and the other five presented for unrelated symptoms when the LCH was diagnosed during screening colonoscopy.

The pathogenesis of LCH is still uncertain and there is a debate on whether it is reactive or neoplastic in nature. Determining the etiology of the disease would help regulate therapy, as each process would have different therapeutic implications. Recent studies have suggested a neoplastic etiology due to the presence of recurrent genetic mutations discovered in LCH cases [6-7]. A study by Badalian-Very et al. revealed that 35 of 61 cases (57%) of LCH contained the BRAF V600E mutation. Another similar study in children showed the same mutation in 63% of the cases that were biopsied [7]. These studies not only provide evidence for the neoplastic nature of LCH but also facilitated the development of targeted therapeutical options. Vemurafenib, a BRAF inhibitor, is being used in patients with severe and refractory cases that are found to have the BRAF V600E mutation [8].

Due to the rare nature of LCH and an incomplete understanding of the pathogenesis, treatment and chemotherapy regimens in adults are not standardized. LCH has been clinically classified into three major groups: unifocal or multifocal involvement of a single organ system and multisystem LCH (where two or more organ systems are affected) [1]. This classification is the basis of risk stratification when trying to determine an appropriate chemotherapeutic regimen. In patients with predominantly single organ involvement, the therapy of choice is based on the organ and number of lesions present. The objective of treatment is to minimize toxicity, and options such as vinblastine and prednisone have been used successfully with curettage/excision of lesions as necessary [9]. Patients with multiple organ involvement should be encouraged to partake in a clinical trial with an initial induction of six months chemotherapy with cytarabine, vinblastine, and prednisone [9]. Due to the lack of data available to guide the treatment of LCH, the course of disease and prognosis vary on a case-by-case basis. Generally, patients with a multifocal and multisystem disease face unfavorable outcomes.

## Conclusions

In summary, LCH involving the GI tract in adults is very rare and usually presents as a localized and incidental finding. LCH involving the GI tract should be considered as a differential diagnosis when polyps or nodular lesions are witnessed on screening colonoscopies. In addition, the lesions must be biopsied to confirm the diagnosis of LCH and additional follow-up is essential to rule out systemic disease.
